# Exploring the Immunopathogenesis of Viral Hemorrhagic Fever in Mice with a Humanized Immune System

**DOI:** 10.3389/fimmu.2017.01202

**Published:** 2017-09-26

**Authors:** Günther Schönrich, Martin J. Raftery

**Affiliations:** ^1^Institute of Medical Virology, Charité – Universitätsmedizin Berlin, Berlin, Germany

**Keywords:** viral hemorrhagic fever, humanized mice, mice with a humanized immune system, virus-induced immunopathogenesis, viruses

## Abstract

Viral hemorrhagic fever (VHF) as a disease entity was first codified in the 1930s by soviet scientists investigating patients suffering from hantavirus infection. The group of hemorrhagic fever viruses (HFVs) has since expanded to include members from at least four different virus families: *Arenaviridae, Bunyaviridae, Filoviridae*, and *Flaviviridae*, all enveloped single-stranded RNA viruses. After infection, the natural hosts of HFVs do not develop symptoms, whereas humans can be severely affected. This observation and other evidence from experimental data suggest that the human immune system plays a crucial role in VHF pathogenesis. For this reason mice with a human immune system, referred to here as humanized mice (humice), are valuable tools that provide insight into disease mechanisms and allow for preclinical testing of novel vaccinations approaches as well as antiviral agents. In this article, we review the impact of humice in VHF research.

## Introduction

Emerging viral hemorrhagic fever (VHF) refers to a group of distinct but similar zoonotic diseases induced by different enveloped RNA viruses. They cause increased vascular permeability that affects one or more organ systems and finally may result in life-threatening shock (1). Thrombocytopenia, another key symptom of VHF, can be due to either increased platelet destruction or decreased platelet production by megakaryocytes (2). Hemorrhagic fever viruses (HFVs) belong to four separate virus families: *Flaviviridae, Bunyaviridae, Arenaviridae*, and *Filoviridae*. Small mammals such as rodents and bats are the natural hosts, which are chronically infected without developing obvious symptoms. Humans are dead-end hosts that usually clear the virus after incidental infection but may develop acute symptoms.

Suitable animal models that reproduce key symptoms of VHF are rare (3–5). Non-human primates (NHPs) are the gold standard for some VHF types such as Ebola virus disease (EVD) but cannot be used for others such as dengue fever (DF) (6, 7). In addition, ethical and economic considerations clearly restrict research with NHPs. Guinea pigs or hamsters show typical symptoms after infection with some HFVs (8–10). However, the lack of species-specific immunological reagents complicates experiments. Laboratory mice often do not support replication of HFV or require the adaption of virus isolates to the mouse, thereby reducing their value as a model of human infection (11, 12).

The advent of humanized mice (humice) has opened up a new avenue for VHF research. In the 1980s, experiments demonstrated successful engraftment of human hematopoietic stem cells (HSCs) in immunodeficient mice (13). Today humice offer the opportunity to gain new and exciting insights into important human diseases such as cancer, allergies, and infections (14–17). Humice are an especially valuable test bed for HFVs. Firstly, HFVs specifically target human myeloid cells such as dendritic cells (DCs) (18–24). Secondly, evidence is accumulating that an inadequate immune response substantially contributes to VHF pathogenesis (25). This aspect is difficult to study in conventional animal models, as their immune system differs substantially due to evolution driven by exposure to different groups of pathogens over millions of years (26–28). For instance, there are major differences regarding the response of pattern recognition receptors to stimulation by invading pathogens. Although closely related to humans, even NHPs show interspecies immunological differences to humans (29, 30).

In this review, we summarize the novel insights gained from experiments with humice in VHF research.

## Categories of Humice Used in VHF Research

The utility of immunodeficient mice as recipients of a human immune system has continuously increased. Efficient reconstitution with human hematopoietic cells was first described in non-obese diabetic (NOD)/severe combined immunodeficiency (SCID) mice (31, 32). The homozygous SCID mutation impairs murine T and B cell development, whereas the NOD background results in deficient natural killer (NK) cell function. The *Sirpa* gene polymorphism in the NOD background also curtails phagocytosis of engrafted human HSCs (33). NOD/SCID mice have subsequently been improved by truncation or deletion of the murine IL-2 receptor common gamma (IL-2Rγ) chain (34–36). This molecule represents an important component of the high-affinity receptors for several inflammatory cytokines. The NOD/SCID/IL-2Rγ^−/−^ (NSG) mice are thus severely deficient in innate immunity and show augmented human HSC engraftment. The reconstitution with human HSCs in NSG mice is long lasting (37). In another approach, the IL-2Rγ^−/−^ mutation was introduced into mice with a mutated recombination activating gene 2 (*Rag2*) on a BALB/c background (38). The *Rag2* mutation in these BALB/c Rag2^−/−^/IL-2Rγ^−/−^ (BRG) mice renders them completely free of murine T and B cell cells, whereas the SCID mutation is “leaky,” meaning that some functional murine T and B cells develop (39).

**Figure 1:**
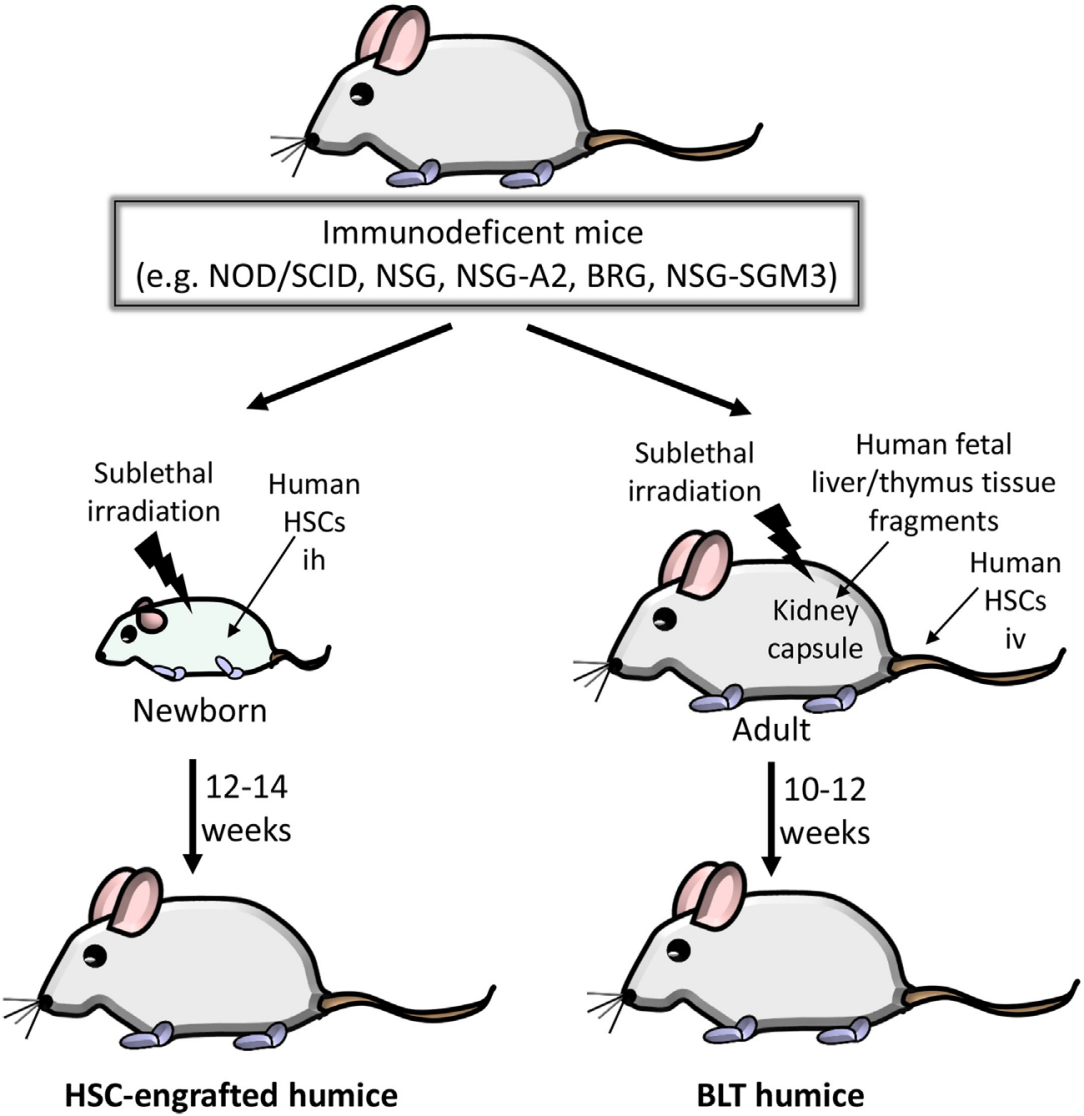
Generation of humice in viral hemorrhagic fever research. Various immunodeficient mice can be used as a platform for generating mice with a human immune system. Non-obese diabetic (NOD)/severe combined immunodeficiency (SCID) mice show impaired murine T and B lymphocyte development due to the homozygous SCID mutation and are in addition deficient in natural killer (NK) cell function due to the NOD background. The *Sirpa* gene polymorphism in the NOD background also blunts phagocytosis of engrafted human hematopoietic stem cells (HSCs). The truncation or deletion of murine IL-2 receptor common gamma (IL-2Rγ) in NOD/SCID/IL-2Rγ^−/−^ (NSG) mice further increases human HSC engraftment. NSG/A2 mice express human leukocyte antigen A2 to facilitate the development of functional CD8 T cells. In BALB/c Rag2^−/−^/IL-2Rγ^−/−^ (BRG) mice, the IL-2Rγ^−/−^ mutation was introduced into BALB/c mice deficient in the recombination activating gene 2 (*Rag2*). Finally, NSG/SGM3 mice allow better development of human myeloid cells due to constitutive expression of human cytokines (stem cell factor, granulocyte/macrophage colony-stimulating factor 2, and IL-3). Left: HSC-engrafted humice. Human HSCs (derived from various sources such as bone marrow, cord blood, peripheral blood or fetal liver) are inoculated intrahepatically (ih) into sublethally irradiated newborn mice. Approximately 12–14 weeks after HSC inoculation, humice are monitored for engraftment of human HSCs by flow cytometric analysis. Right side: bone marrow/liver/thymus (BLT) humice. Human fetal liver and thymus are transplanted under the kidney capsule of sublethally irradiated 6- to 8-week-old mice and subsequently inoculated iv with autologous human fetal liver HSCs. The engraftment is verified 10–12 weeks later.

The different types of humice differ with regard to efficiency of human HSC engraftment and the resulting composition of human hematopoietic cells (40–42). In VHF research, mainly HSC-engrafted humice and bone marrow/liver/thymus (BLT) humice are used. In the HSC-engrafted humice, human CD34^+^ HSCs from various sources (bone marrow, cord blood, peripheral blood or fetal liver) are inoculated into newborn immunodeficient mice and allowed to develop (Figure 1). A major disadvantage of HSC-engrafted humice is the lack of human T cell education due to the absence of a human thymus. This situation has been improved by generating transgenic NSG mice expressing human leukocyte antigen (HLA) molecules. Transgenic NSG mice expressing the HLA class I molecule HLA-A2 (hereafter referred to as NSG-A2 mice) facilitate the development of functional CD8 T cells after reconstitution with HLA-A2^+^ human HSCs (43–45). The expression of HLA class II molecules allows the development of both antibody-producing and class-switching human B cells (46–48).

The BLT humice enables human T cells to differentiate in an autologous human thymus (49, 50). BLT mice are generated by transplantation of human fetal liver and thymus tissue fragments under the kidney capsule of immunodeficient mice, e.g., NOD/SCID or NSG mice, followed by intravenous injection of autologous HSCs derived from fetal liver (Figure 1). The major advantage of BLT mice is their ability to mount a relatively effective human adaptive immune response due to the presence of a human thymic environment and the resultant HLA-restricted T cell repertoire. Caveats are the requirement of human fetal tissue and the relatively frequent development of graft-versus-host disease.

Elimination of human hematopoietic cells by murine phagocytic cells combined with defective human hematopoiesis in humice put a curb on human erythrocytes (51, 52), platelets (53), neutrophils (54–56), monocytes/macrophages (57), and NK cells (58, 59). An explanation for defective human hematopoiesis is the lack of binding of important murine growth factors and cytokines to receptors on human progenitor cells. An elegant solution of this problem is the generation of homozygous knock-in mice to replace murine with human cytokines (60–63). Germline-competent ES cells from NSG mice have been established to facilitate their genetic modification (64). Recently, transgenic NSG mice have been developed that constitutively express human “myeloid” cytokines: human stem cell factor, human granulocyte/macrophage colony-stimulating factor 2, and human IL-3. After reconstitution with human HSCs, these NSG-SGM3 mice allow better development of human myeloid cells, the key target cells of VHF viruses (65–68).

So far, four different HFVs from three virus families (*Flaviviridae, Filoviridae*, and *Bunyaviridae*) have been studied in humice.

## Flaviviruses

Dengue viruses (DENVs) are the cause of the most important arthropod-borne viral disease in terms of global distribution and economic impact (69). The known DENV serotypes (DENV-1 to DENV-4) are members of the *Flaviviridae* family and carry a positive-sense single-stranded RNA genome. The *Aedes aegypti* mosquito, which is found in tropical and subtropical areas, functions as the main vector. Roughly 2.5 billion people, i.e., two fifths of mankind, live in endemic areas. An estimated 390 million people become infected per year. The most frequent clinical manifestation is DF, a self-limiting febrile disease with spontaneous recovery (70). However, some patients develop major complications such as plasma leakage leading to shock, respiratory distress, bleeding and organ impairment.

DF has been extensively studied in humice (Table 1). After DENV-2 infection, NOD/SCID mice and NSG mice develop fever, erythema, and human thrombocytopenia compatible to the human disease (71–73). The decrease in human platelets is due to inhibition of human megakaryocyte development (74). DENV-2 could be detected in several human cell types in the bone marrow, spleen, and blood of these mice (73). In accordance, human cells isolated from the bone marrow of NSG mice were susceptible to DENV-2 infection *in vitro* (43). This cell tropism is in agreement with studies demonstrating DENV-derived protein in phagocytic cells in human autopsy tissue such as lymph nodes and spleen (75). Intriguingly, when infected *Aedes aegypti* transmitted DENV-2 to humice during feeding, more sustained and severe viremia, erythema and thrombocytopenia occurred compared to other modes of virus inoculation (76). This suggests that the mosquito bite itself and mosquito saliva contribute to dengue pathogenesis.

**Table 1:** Humanized mouse models in viral hemorrhagic fever (VHF) research.

Disease	Virus/family	Platform	Key findings	Reference
DF	DENV-2/*Flaviviridae*	NOD/SCID, NSG	DF symptoms (fever, rash, and thrombocytopenia)	(71, 72)
DF	DENV-2/*Flaviviridae*	NSG	DENV-2 tropism as in human DF	(43, 73)
DF	DENV-2/*Flaviviridae*	NSG	Thrombocytopenia due to inhibition of megakaryocyte development	(74)
DF	DENV-2/*Flaviviridae*	NOD/SCID-BLT, NSG	Effective DF treatment with adenosine nucleoside inhibitor or therapeutic antibody	(84, 85)
DF	DENV-2/*Flaviviridae*	NSG/A2	Virus-specific HLA-A2-restricted human T cell response	(43)
DF	DENV-2/*Flaviviridae*	BRG, NSG, NSG/A2	Virus-specific huIgG and huIgM response	(43, 76, 78)
DF	DENV-2/*Flaviviridae*	BLT-NSG	Serotype-cross-reactive huIgM antibodies with poor neutralizing activity	(80, 81)
DF	DENV-2/*Flaviviridae*	NSG/SGM3-BLT	Higher levels of antigen-specific huIgM and huIgG compared to BLT-NSG	(82)
DF	DENV-2/*Flaviviridae*	NSG	Serum metabolomics similar to human DENV infections	(83)

EVD	EBOV/*Filoviridae*	NSG-A2	EVD symptoms (cell damage, liver steatosis, hemorrhage, high lethality)	(96)
EVD	EBOV/*Filoviridae*	NSG-BLT	Increased levels of pro-inflammatory cytokines and liver enzymes; histopathological findings typical for EVD	(94)
EVD	EBOV/*Filoviridae*	NSG-SGM3	Absence of characteristic EVD histopathology	(95)

CCHF	CCHFV/*Bunyaviridae*	NSG-SGM3	Lethal disease with severe neuropathology (gliosis, meningitis, meningoencephalitis)	(99)

HFRS	HTNV/*Bunyaviridae*	NSG, NSG-A2	Highest numbers of HTNV copies in the lung, humanized NSG-A2 mice develop faster and more severe symptoms such as thrombocytopenia	(112)

*BLT, bone marrow/liver/thymus model; BRG, BALB/c Rag2^−/−^ IL-2Rγ^−/−^ mice; CCHF, Crimean–Congo hemorrhagic fever; CCHFV, Crimean-Congo hemorrhagic fever virus; DENV-2, dengue virus serotype 2; DF, dengue fever; EBOV, Ebola virus; EVD, Ebola virus disease; HFRS, hemorrhagic fever with renal syndrome; HTNV, hantaan virus; NOD, non-obese diabetic mice; NSG, NOD/SCID/IL-2Rγ^−/−^ mice; NSG-A2, NSG mice constitutively expressing HLA-A2; NSG-SGM3, NSG mice constitutively expressing human stem cell factor, human granulocyte/macrophage colony-stimulating factor 2, and human IL-3; SCID, severe combined immunodeficiency mice; HLA, human leukocyte antigen*.

The immune system plays a crucial role in dengue pathogenesis (25, 77). Firstly, in humans, priming of the antiviral immune response with one DENV serotype often causes a more severe disease after infection with another DENV serotype at a later time point. Secondly, the most severe symptoms are observed at the peak of the human antiviral immune response. For these reasons the response of human immune cells has been studied in humice of DENV infection. Human anti-DENV IgM antibodies were detected 2 weeks after infection of BRG mice with DENV-2 followed by virus-reactive IgG at 6 weeks postinfection (78). In accordance, it was observed that NSG mice infected with DENV-2 through mosquito bite developed a virus-specific adaptive immune response (76). Moreover, human T cells from infected NSG-A2 mice secreted cytokines in response to known stimulatory HLA-A2-restricted DENV-2 peptides (43). Finally, NK cells are activated by contact with infected DCs before they control DENVs through IFN-γ secretion (79).

The virus-specific immune response has also been studied in DENV-2-infected NSG-BLT mice (80, 81). Human T cells isolated from NSG-BLT mice during acute infection and in the convalescence phase secreted IFN-γ after stimulation with DENV-2 peptides (80). In addition, human B cells secreted DENV-2-reactive IgM antibodies (80). The majority of these antibodies were serotype cross-reactive, recognized epitopes on envelope proteins and intact virions, and neutralized poorly (81). The antibodies generated in the convalescence phase showed higher avidity compared to antibodies found in acute infection (81). Accordingly, NSG-BLT mice in the convalescence phase showed decreased virus titers after being challenged with a clinical DENV-2 strain. Furthermore, preincubation of DENV-2 virions with immune sera from immune NSG-BLT mice reduced viral replication after inoculation into naïve mice (81). In DENV-2-infected BLT mice generated from NSG-SGM3 mice, improved B cell development and higher levels of antigen-specific IgM and IgG were observed compared to DENV-2-infected NSG-BLT mice (82). The serum metabolomics of DENV-2-infected humice is similar to human DENV infections demonstrating the utility of humice for analyzing DENV-associated pathogenesis (83). In addition, a therapeutic antibody and an antiviral drug were successfully tested in DENV-2-infected humice (84, 85). These studies emphasize the value of humice in translational and preclinical VHF research.

## Filoviruses

The dramatic 2014 outbreak of EVD in West Africa underlines the need to better understand this deadly disease (86). Ebola virus (EBOV) and Marburg virus, a closely related HFVs, belong to the *Filoviridae* family in the order *Mononegavirales* (87). These large enveloped filamentous viruses are equipped with a negative-sense single-stranded RNA genome. Bats represent potential reservoirs for Marburg virus (88) and, more speculatively, perhaps also EBOV. They are persistently infected without showing symptoms and can spread the viruses to humans and NHPs. EVD has a high case fatality rate and affects many organs resulting in a variety of symptoms including gastrointestinal, respiratory, neurological, and vascular (89). Most impressive are the hemorrhagic manifestations such as petechiae, ecchymoses, and mucosal hemorrhages. The final and most severe stage of EBOV disease is characterized by shock, systemic impairment of coagulation and convulsions. The fatal outcome is most likely a consequence of both the direct effects of lytic EBOV replication and an inadequate immune response (90, 91). In EVD survivors, long-lasting activated CD8 T cells have been detected, suggesting that EBOV-derived stimulatory antigen persists at low levels within the organism (92).

Small animal models for analyzing filovirus pathogenesis have been generated using laboratory mice, guinea pigs, and the Syrian hamster (93). Recently, the potential of humice for modeling EBOV disease was explored in three different types of humice (Table 1) (6, 94–96). To this end, NSG-A2, NSG-SGM3, and NSG-BLT mice were infected with low-passage wild-type EBOV isolates. EBOV-infected NSG-A2 mice started to lose weight around day 7 postinfection and some hallmarks of human EBOV disease were observed including cell damage, liver steatosis, signs of hemorrhage, and high lethality (96). Intriguingly, there was a direct correlation between EBOV disease severity and the level of HSC engraftment. In contrast, unreconstituted NSG-A2 mice showed only mild symptoms with weight loss starting later in the third week postinfection and gradually continuing until the time of death around day 30 postinfection. NSG-A2 mice reconstituted with normal murine HSCs, another important control, survived EBOV infection. These results emphasize the importance of human hematopoietic cells for EVD pathogenesis.

In EBOV-infected NSG-BLT mice, clinical illness depended on viral dose inoculated and donor tissue used for reconstitution (94). Moderate leukopenia and thrombocytopenia and histopathological alterations similar to those found in human victims were observed. Liver enzymes and key pro-inflammatory human cytokines associated with fatal EVD (e.g., TNF-α, IL-1, IL-6, and IL-10) were increased. In contrast, unreconstituted NSG control mice survived EBOV, underlining the role of human hematopoietic cells in EVD pathogenesis.

After EBOV infection of NSG-SGM3 mice, high virus titers were found in blood, liver, and spleen (95). Most of the mice died within 2 weeks of infection. In accordance with the concept that human myeloid cells spread VHF viruses within the organism, viral antigen was found in tissue-residing human macrophages and DCs and later in the course of infection also in murine parenchymal cells. In contrast to EBOV-infected NSG-A2 and NSG-BLT mice, the characteristic histopathology of severe human EBOV disease was not observed. This difference could be explained at least in part by the lack of HLA class I-restricted functional T cells in NSG-SGM3 mice. Thus, the lethal disease observed in these mice may be due to pathology directly induced by EBOV or due to innate immune responses.

## Bunyaviruses

A number of HFVs belong to the family *Bunyaviridae*. These are enveloped viruses that carry a genome consisting of three negative-sense single-stranded RNA segments (97). Recently, Crimean-Congo hemorrhagic fever virus (CCHFV) belonging to the genus *Nairovirus* and Hantaan virus (HTNV), the prototype member of the genus *Hantavirus*, have been analyzed in humice.

Crimean–Congo hemorrhagic fever (CCHF) represents the most relevant tick-borne viral disease in humans due to its wide distribution. Sporadic cases or outbreaks of CCHF are observed in a vast geographic area including western China, the Middle East, southern Europe, and most parts of Africa (98). CCHFV circulates in wild and domestic vertebrates that are transiently infected without showing symptoms. Humans become infected through tick bite or contact with body fluids from infected patients or animals. As with other VHFs, the spectrum of symptoms of Crimean-Congo hemorrhagic fever includes mild fever, vascular leakage resulting in multiorgan failure, and finally shock with coagulation defects. Case fatality rates of up to 30% have been reported. A recent study analyzed CCHFV-infected NSG-SGM3 mice (Table 1) (99). They showed lethal disease resembling CCHF in some respects. CCHFV was detected in many organs including liver, spleen, and brain, similar to CCHFV-infected mice deficient in type I IFN responses. Histopathological analysis revealed several features typically found in CCHF such as the presence of viral antigen within Kupffer cells, endothelial cells, and hepatocytes. Similar to human CCHF cases, vacuolar degeneration/steatosis and increased single cell necrosis were observed. CCHV-infected humice also developed CNS symptoms such as meningitis and meningoencephalitis. Intriguingly, a population of activated human CD8 T cells was identified that could contribute to immunopathology or virus elimination in a non-specific (HLA class I-independent) way (99).

Hantaviruses are globally emerging pathogens responsible for VHF in Africa, America, Asia, and Europe (100). Rodents, shrews, moles, and bats serve as natural hosts for hantaviruses. In contrast to all other pathogenic members of the family *Bunyaviridae*, hantaviruses are transmitted to humans *via* aerosols derived from rodent excreta. Depending on the geographic region, hemorrhagic fever with renal syndrome (HFRS) or hantavirus cardiopulmonary syndrome (HCPS) may develop (101). Both types of disease bear pathogenic similarities with increased vascular permeability and loss of platelets as leading symptoms (102). Hantavirus replicate in cell culture without causing obvious cytopathic phenomena, suggesting that immune mechanisms play a role in HFRS/HCPS (103, 104). In line with this view, the susceptibility to hantavirus infection and the clinical course of hantavirus-induced disease in humans are linked to polymorphisms of immune-related genes (105). Moreover, pathogenic hantaviruses infect human myeloid cells such as DCs and monocytes and interact with neutrophils, the most abundant immune cells (21, 23, 106–109). This tropism may help the pathogens to spread within the organism. In addition, this may also result in an inadequate immune response such as the excessive release of neutrophil extracellular traps that damages the endothelial barrier (110, 111).

Recently, hantaviral pathology was analyzed in HTNV-infected NSG mice and NSG-A2 (Table 1) (112). In both types of humice, hantaviral genomic RNA was detected in the kidney, liver, and spleen, but the highest viral copy numbers were found in the lung. Significant weight loss occurred earlier in NSG-A2 mice (day 10) than in NSG mice (day 15). HTNV-infected unreconstituted NSG mice that served as a control showed only a slight but not significant weight loss within the observation period. Inflammatory infiltrates in the lung of HTNV-infected NSG-A2 mice were stronger than in NSG mice. Similarly, the number of human platelets dropped significantly in NSG-A2 mice, whereas the observed reduction in NSG mice was not significant. Although hantaviruses infect human megakaryocytic cells, they do not cause alterations in cell survival or differentiation (113). Thus, it is likely that hantavirus-induced thrombocytopenia is due to increased platelet destruction (114). Taken together, these findings indicate that human hematopoietic cells including HLA-A2 restricted human T cells play a pivotal role in hantaviral pathogenesis.

## Conclusion and Future Directions

Humice are an extremely useful but still not optimal tool for elucidating the mechanisms of VHF immunopathogenesis, in particular, because of the very limited range of alternative research models. In addition, humice facilitate testing of vaccines and novel antiviral agents (115). Development of these therapeutic agents is urgently needed for treatment and prevention of highly lethal VHFs. For example, humice can be used to generate human monoclonal antibodies for VHF prophylaxis (116). Finally, standardized humice allow the prospective testing of newly discovered HFVs or viruses suspected to be potentially HFVs and could form part of a zoonosis threat detection network. Future attempts have to improve the utility of humice as VHF models by further allowing better engraftment and differentiation of HSCs as well as the development of a fully functional lymphoid tissue architecture that efficiently supports human immune reactions.

## Author Contributions

Both authors contributed to the conception, writing, and critical revising of this review.

## Conflict of Interest Statement

The authors declare that the research was conducted in the absence of any commercial or financial relationships that could be construed as a potential conflict of interest.
